# Books and Pamphlets Received

**Published:** 1885-03-20

**Authors:** 


### BOOKS AND PAMPHLETS RECEIVED.

*An Epitome of Eye, Ear, Throat and Nose Diseases*, for the Student and Practitioner. Illustrated with wood cuts and photographs. By Arthur G. Hobbs, M.D , Professor of Ophthalmology and Laryngology in the Southern Medical College, Atlanta, Ga. Philadelphia: Samuel M. Miller, M.D., Publisher, 1885.

This is a work of 202 pages of close, condensed, practical matter, touching upon every important and essential point in the departments of which it treats. The author seems well and truly to have accomplished his purpose, as stated in his preface, "To compress within the narrowest limits such a clear and intelligible account of the diseases in question as is essential for the purpose of practical information." Based, as he states, upon an experience of the wants of college students, as well as the needs of the practitioner, the work must prove both timely and acceptable. It is certainly convenient and desirable to the student to have thus placed before him, in the pressure of his labors during a college course, a concise outline and statement of the facts he is required to remember. And it is equally satisfactory to the busy practitioner to have before him an epitome of the leading facts and principles, as contained in the works of a number of our ablest authors upon these difficult and troublesome affections. The author, as a Specialist in these affections, was well qualified for the task which he assumed, and he deserves the thanks of the profession for the able and admirable manner in which he has accomplished it.

We commend the work as eminently practical and useful, and as one which every student of medicine should possess, and which every practitioner should hasten to add to his library.

